# An integrated proximity labeling and vesicle reconstitution assay identifies novel regulators of Sonic hedgehog secretion

**DOI:** 10.1091/mbc.E25-12-0627

**Published:** 2026-05-28

**Authors:** Ziyang Song, Zixin Chen, Ye Tian, Qianyuan Wang, Xiaoxu Zhao, Yusong Guo, Xiao Tang

**Affiliations:** ^a^Anhui Provincial Key Laboratory of Molecular Enzymology and Mechanism of Major Metabolic Diseases, College of Life Sciences, Anhui Normal University, Wuhu 241002, Anhui, China; ^b^Guangdong Cardiovascular Institute, Medical Research Institute, School of Basic Medical Science, Guangdong Provincial People's Hospital (Guangdong Academy of Medical Sciences), Southern Medical University, Guangzhou 510080, Guangdong, China; ^c^Division of Life Science, The Hong Kong University of Science and Technology, Hong Kong 999077, China; ^d^Hong Kong University of Science and Technology Shenzhen Research Institute, Shenzhen 518057, China; MRC Laboratory of Molecular Biology

## Abstract

Protein trafficking is a fundamental process for cellular organization and signaling. However, identifying the specific machinery that packages cargo into transport vesicles has been a significant challenge. Conventional proximity biotinylation methods often fail to distinguish proteins that are merely near a cargo from those that are functionally co-packaged into the same vesicles, leading to high background noise. To address this limitation, we developed an integrated strategy that combines in vivo proximity biotinylation with an in vitro reconstituted vesicle formation assay. Applying this method to the signaling morphogen Sonic hedgehog (Shh), we successfully enriched and identified proteins co-incorporated into Shh-containing vesicles. Comparative proteomics and subsequent functional validation revealed two novel regulators of Shh secretion: ER-Golgi Intermediate Compartment Protein 2 (ERGIC2), which is essential for efficient ER-to-Golgi transport, and Sec1 Family Domain Containing 2 (SCFD2), which is critical for post-Golgi export. This study establishes a generalizable method to map vesicle-associated interactomes and provides a more comprehensive molecular framework for the regulated secretion of this important morphogen.

## Abstract (executive summary)

- A major challenge in studying cargo trafficking is distinguishing proteins that are merely near a secretory cargo from those functionally co–packaged into its transport vesicles.
- To address this, we developed an integrated proximity labeling and in vitro vesicle reconstitution assay that selectively captures proteins co–sorted into budding vesicles.
- Using this platform, we identified ERGIC2 and SCFD2 as novel regulators of Shh secretion functioning at distinct trafficking steps.
- This work provides a generalizable strategy to map cargo–specific vesicular interactomes, offering a new tool for dissecting secretion mechanisms in development and disease.

## INTRODUCTION

The fidelity and efficiency of protein trafficking are fundamental to cellular organization and signaling. In eukaryotic cells, the accurate delivery of cargo proteins from their site of synthesis to their functional destinations depends on a sophisticated vesicular transport system. Within this system, cargoes are selectively packaged into distinct transport vesicles, such as Coat Protein Complex (COP) II, COPI, and clathrin-coated vesicles, that bud from donor membranes and fuse with specific acceptor compartments (Guo *et al.*, 2014). The fidelity of this system relies on specialized molecular machinery, which includes, but is not limited to, cargo receptors that recognize luminal cargoes, cytosolic adaptors that link receptors to vesicle coats, small GTPases that regulate coat assembly and membrane deformation, tethering factors that mediate the initial recognition between vesicles and target membranes, motor proteins and their adaptors that transport vesicles along the cytoskeleton, and Soluble N-ethylmaleimide-sensitive factor attachment protein receptor (SNARE) complexes that catalyze membrane fusion (Yoshihisa *et al.*, 1993; Tang and Guo, 2024).

This regulatory framework is critical for the secretion of key signaling molecules, such as Sonic hedgehog (Shh). As an essential regulator of cell proliferation, differentiation, embryonic patterning, and organogenesis, Shh is synthesized and processed into its active N-terminal fragment (ShhN; Carballo *et al.*, 2018). Although certain factors, such as the cargo receptor Surfeit locus protein 4 (SURF4), glutamate receptor 1 (GRIA1), and proteoglycans (PGs), have been identified to regulate Shh trafficking (Tang *et al.*, 2022; Tang *et al.*, 2025), the broader molecular networks, including other adaptors and regulators involved in its secretion, are still not fully elucidated. Therefore, identifying additional regulatory factors is essential for a comprehensive understanding of the molecular mechanisms governing Shh trafficking and secretion, which is central to deciphering its precise spatiotemporal control in development and disease.

Identifying the specific protein factors that interact with a cargo like Shh during its intracellular transport has been a persistent challenge (Ngounou Wetie *et al.*, 2014). Traditional biochemical methods, such as co-immunoprecipitation, often fail to capture transient or weak interactions within dynamic vesicle populations. Proximity-dependent biotinylation has emerged as a powerful tool for mapping the spatial proteome surrounding a protein of interest (Hung *et al.*, 2016). However, it has a significant limitation for trafficking studies: it cannot distinguish proteins that are merely proximal to the cargo from those that are functionally co-packaged into the same transport vesicles, thereby obscuring the identification of bona fide vesicle-associated transport factors.

To overcome this limitation, we developed an integrated strategy that combines the proximity biotinylation with an in vitro reconstituted vesicle formation assay. Our method involves fusing the engineered peroxidase Ascorbate Peroxidase 2 (APEX2) to ShhN. Following performing proximity biotinylation in live cells to label neighboring proteins, we employ a well-established cell-free system to generate vesicles from semipermeabilized cells (Tang *et al.*, 2020). This step selectively isolates only those biotinylated proteins that are co-packaged with ShhN into transport vesicles, thereby effectively excluding the background proteins from static organelles. Applying this combined assay, we performed comparative proteomics under conditions that support or inhibit vesicle formation. This design allowed us to map a high-confidence set of ShhN-associated vesicular interactors. Subsequent functional validation revealed novel regulators of ShhN secretion: ER-Golgi Intermediate Compartment Protein 2 (ERGIC2), which is required for ER-to-Golgi transport, and Sec1 Family Domain Containing 2 (SCFD2), which is essential for post-Golgi vesicular export. This work establishes a generalizable method for identifying novel molecular components in the secretory pathway of a critical morphogen.

## RESULTS

### Combination of proximity biotinylation and in vitro reconstituted vesicle formation assay for proteomic analysis

**FIGURE 1:**
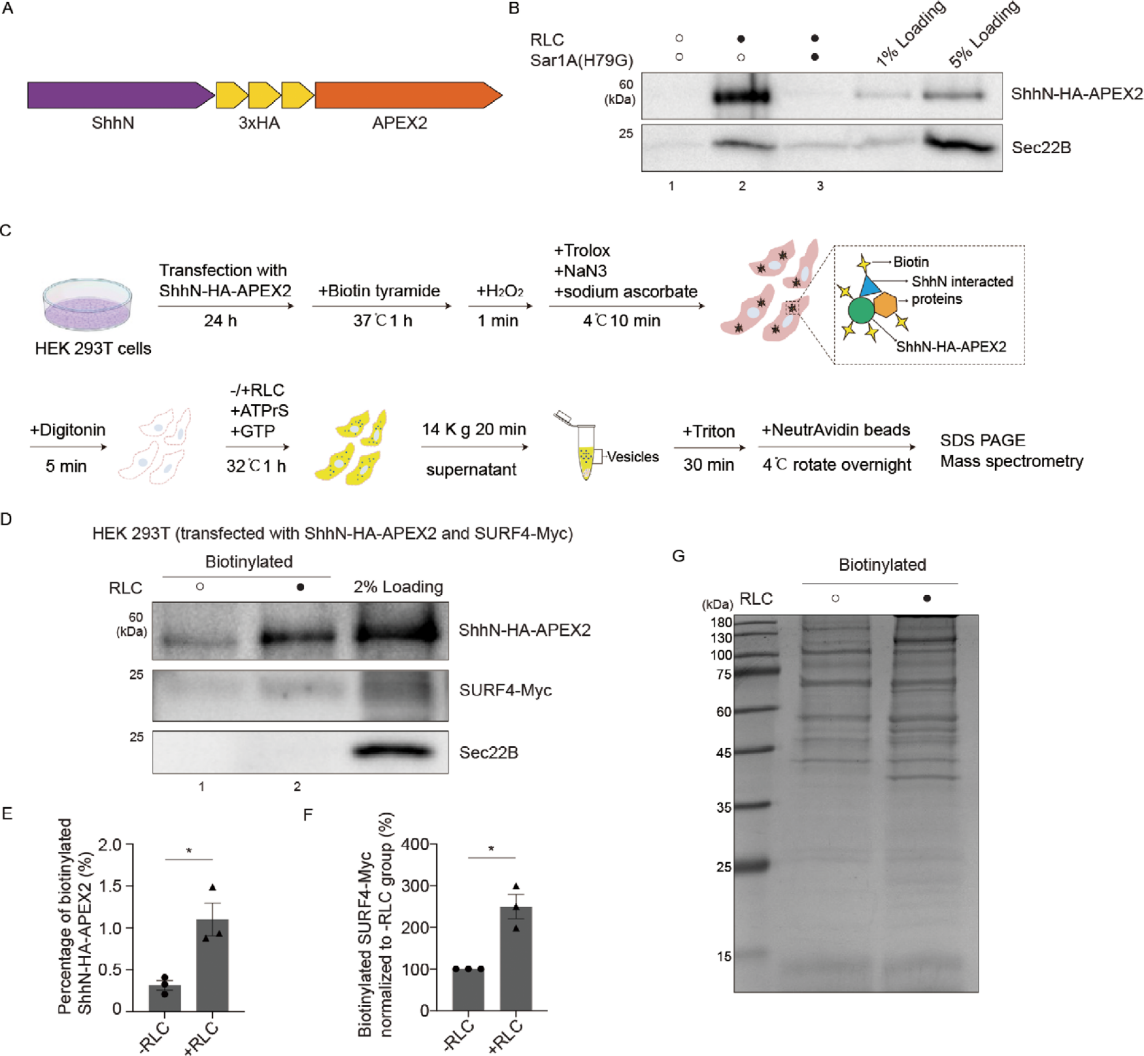
A proximity biotinylation and vesicle formation combined assay for proteomic analysis. (A) Diagram depicting the construction of ShhN-HA-APEX2. (B) HEK293T cells were transfected with plasmids encoding ShhN-HA-APEX2. Twenty four hours after transfection, vesicle formation was performed using the indicated reagents. The vesicle fraction was analyzed by immunoblotting using anti-Sec22B or anti-HA antibodies. (C) Diagram depicting the proximity biotinylation and vesicle formation combined assay for proteomic analysis. (D and G) HEK293T cells were co-transfected with plasmids encoding ShhN-HA-APEX2 and SURF4-Myc. Twenty four hours after transfection, proximity biotinylation and vesicle formation assay were performed in absence or presence of RLC. The biotinylated proteins in the vesicles were analyzed by SDS–PAGE and immunoblotting using anti-Sec22B, anti-Myc or anti-HA antibodies (D) or Coomassie blue staining (G). (E) Relative levels of biotinylated ShhN-HA-APEX2 that bound to NeutrAvidin beads were quantified (*n* = 3, mean ± SD). The quantification is normalized to the level of semi-intact input. *P < 0.05. (F) Relative levels of biotinylated SURF4-Myc that bound to NeutrAvidin beads were quantified (*n* = 3, mean ± SD). The quantification is normalized to the level in −RLC group. *P < 0.05.

Proximity biotinylation utilizes an engineered biotin ligase fused to a protein of interest to label proximal proteins, enabling the mapping of protein–protein interactions. However, proximity biotinylation alone cannot conclusively identify functionally relevant vesicle-associated interactors. To break through this limitation, we combined proximity biotinylation with an in vitro vesicle formation assay to specifically capture the protein candidates that are co-enriched with cargo protein, Shh, in transport vesicles. ShhN was fused to a 3 × HA tag and the engineered peroxidase, APEX2 (ShhN-HA-APEX2; Figure 1A). We first verified that this construct is competent for vesicle packaging. HEK 293T cells expressing ShhN-HA-APEX2 were used to perform an in vitro vesicular formation assay as previously described (Tang *et al.*, 2020). Briefly, the cells were permeabilized by digitonin for 5 min, and the semi-intact cells were incubated at 32°C for 1 h with ATP regeneration system (ATPrS) and GTP in the presence or absence of rat liver cytosol (RLC). The released vesicles were collected by centrifugation and analyzed by immunoblotting. We found that ShhN-HA-APEX2 was enriched in vesicles in an RLC-dependent manner (Figure 1B, comparing lanes 1 and 2). Inclusion of a GTP hydrolysis-defective mutant Sar1A (H79G) strongly impaired packaging of both canonical COPII cargo, Sec22B, and ShhN-HA-APEX2 (Figure 1B, comparing lanes 2 and 3).

We then performed proximity biotinylation experiments using HEK 293T cells transfected with ShhN-HA-APEX2. Upon the addition of hydrogen peroxide to cells preloaded with biotin tyramide substrate, APEX2 generates biotin-phenoxyl radicals that covalently tag proximal proteins. We then performed the vesicle formation assay to collect the vesicle fraction. The biotinylated proteins in the vesicle fraction were lysed and isolated by incubation with NeutrAvidin beads (Figure 1C). As SURF4 was previously established as an ER cargo receptor of Shh, we used SURF4 as a positive interactor in our assay. HEK 293T cells co-expressing ShhN-HA-APEX2 and Myc-tagged SURF4 (SURF4-Myc) were processed through the combined proximity-labeling and vesicle-formation workflow. Both ShhN-HA-APEX2 and SURF4-Myc were biotinylated in the vesicle fraction, while Sec22B was not (Figure 1D, comparing lanes 1 and 2, and quantification in 1E and 1F). Notably, endogenous SURF4 was also biotinylated in the +RLC condition (Supplemental Figure S1A, lane 2), further verifying the specificity and feasibility of our assay. To assess the purity of our vesicle preparation, we examined the ER marker Calnexin. The Calnexin signal in both the biotinylated and vesicle fractions was negligible compared with the semi-intact cell input (Supplemental Figure S1A; comparing lanes 2, 5 with 3). Importantly, unlike ShhN-HA-APEX2 and SURF4-Myc, Calnexin showed no significant enrichment in the +RLC condition relative to the −RLC control (Supplemental Figure S1A, comparing lanes 1 and 2, 4 and 5). These results indicate that the observed RLC-dependent enrichment is specific to vesicle-associated cargo proteins rather than nonspecific membrane fragments.

To identify vesicle-associated proteins biotinylated by ShhN-HA-APEX2, we performed the assay in HEK293T cells expressing ShhN-HA-APEX2 under two conditions, in the presence (+RLC) or absence (−RLC) of rat liver cytosol. The biotinylated proteins were then analyzed by SDS–PAGE and Coomassie blue staining (Figure 1G). The proteins were then in-gel digested with trypsin and analyzed by label-free quantitative mass spectrometry with three biological repeats.

### Bioinformatic mapping of ShhN-associated vesicular interactors

**FIGURE 2:**
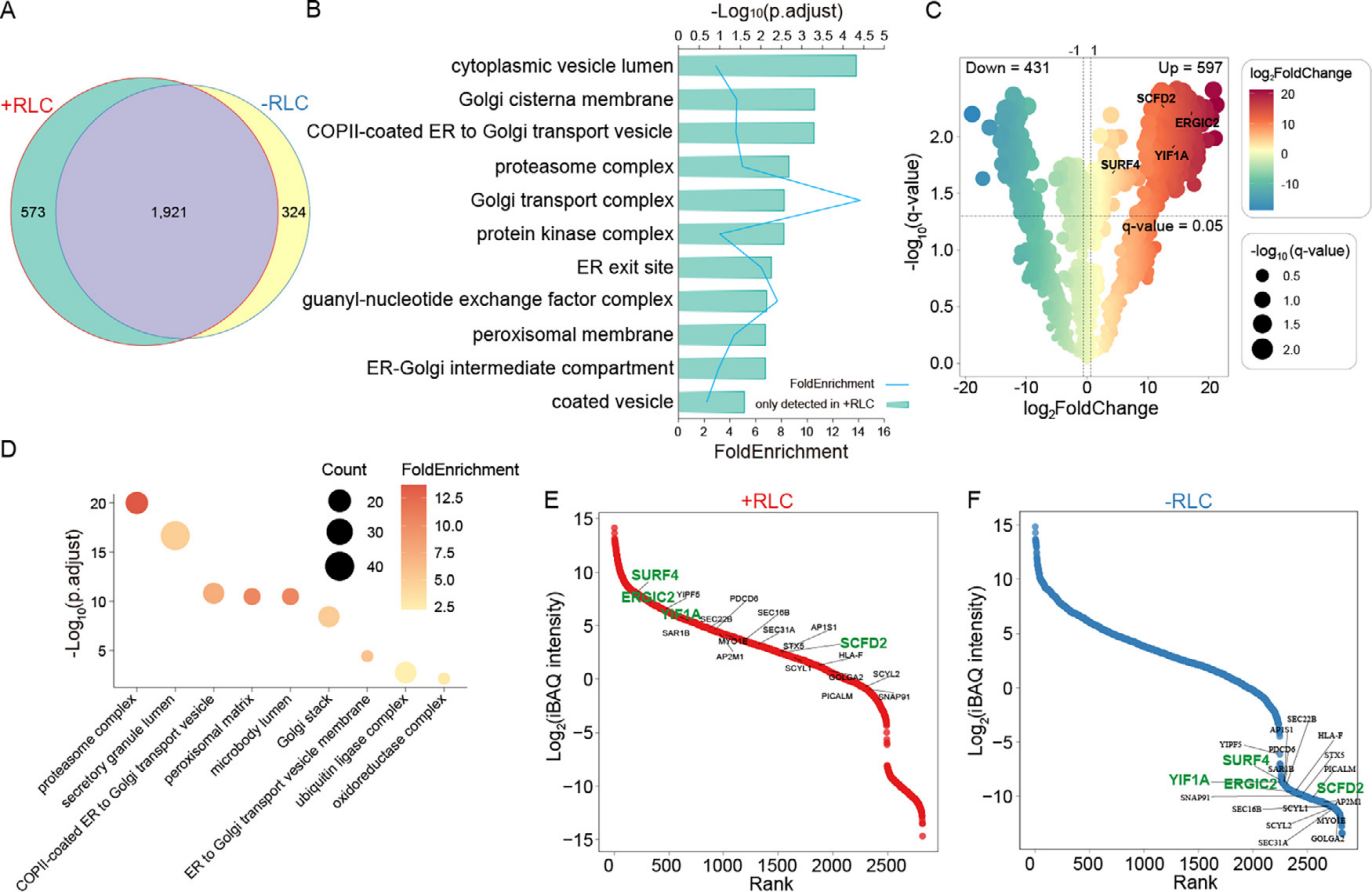
Bioinformatic mapping of ShhN interactors in transport vesicles. (A) A Venn diagram depicting the intersection of biotinylated proteins in the vesicles formed in the −RLC or +RLC groups. (B) GO cellular component enrichment analysis of proteins uniquely detected in the +RLC. (C) Volcano plot illustrating differentially expressed biotinylated proteins in the +RLC group compared with the −RLC group. Proteins with a *q*-value < 0.05 and |log₂(fold change)| > 1 were considered statistically significant. (D) GO cellular component enrichment analysis of up-regulated biotinylated proteins in the +RLC condition. (E and F) Cumulative abundance curves ranking biotinylated proteins in the +RLC (E) and −RLC (F) groups. Proteins involved in coated vesicles are highlighted.

The proteomic analysis identified 2494 proteins in the +RLC group and 2245 proteins in the −RLC group (Supplemental Table S1), with 1921 common to both groups. 573 proteins were exclusive to the +RLC group, and 324 were exclusive to the −RLC group. (Figure 2A). Gene Ontology (GO) enrichment analysis revealed that +RLC-unique proteins were significantly associated with secretory compartments, including Golgi cisterna membranes, COPII-coated ER-to-Golgi transport vesicles, ER exit site, and ER-Golgi intermediate compartment (Figure 2B). Quantitative differential expression analysis further supported this compartmental specificity. Compared with the −RLC control, the +RLC condition showed significant enrichment of 597 proteins (Figure 2C; Supplemental Table S2). Cellular component analysis confirmed that the enriched proteins were predominantly associated with secretory compartments (Figure 2D). To prioritize candidates potentially involved in Shh trafficking, we ranked biotinylated proteins by their relative abundance, a proxy for interaction strength, and highlighted those associated with coated vesicles. This analysis confirmed a stronger association of these vesicular proteins with ShhN in the +RLC condition, as shown in the cumulative abundance curves for the +RLC (Figure 2E) and −RLC (Figure 2F) groups. Notably, the previously reported Shh interactor SURF4 was also identified among the high-ranking candidates (Figure 2, E and F), further validating the reliability of the dataset.

### Identification and validation of ShhN interactors that impair ShhN secretion

To assess the functional relevance of the identified vesicle-associated interactors in ShhN trafficking, we selected three high-ranking candidates, ER-Golgi Intermediate Compartment Protein 2 (ERGIC2), Sec1 Family Domain Containing 2 (SCFD2), and Yip1 Interacting Factor Homolog A (YIF1A), for loss-of-function studies (Figure 2, E and F). ERGIC2 is a COPII-associated protein known to regulate the transport of gap junction proteins from the ER to the Golgi (Guan *et al.*, 2022). Its yeast ortholog, ER-derived vesicle protein 41 (Erv41), functions as a dual-role cargo receptor. It facilitates the anterograde transport of specific Golgi-resident mannosyltransferases (Noda *et al.*, 2014) and mediates the retrograde retrieval of escaped ER proteins (Shibuya *et al.*, 2015). SCFD2 is a member of the Sec1/Munc18 (SM) protein family. Members of the same protein family, such as SCFD1, have been shown to play key roles in SNARE-complex assembly (Eisemann *et al.*, 2020). Although genomic and transcriptomic studies have linked SCFD2 to diverse biological processes, including spermatogenesis (Ding *et al.*, 2025), neural developmental (Tang *et al.*, 2019; Gupta *et al.*, 2023), and hormone-dependent breast cancer pathogenesis (Takeiwa *et al.*, 2022), its precise molecular mechanisms remain poorly characterized. YIF1A is an ER and Golgi recycling protein that participates in membrane delivery (Kuijpers *et al.*, 2013) and maintains ER and Golgi structure (Yoshida *et al.*, 2008; Dykstra *et al.*, 2013).

**FIGURE 3:**
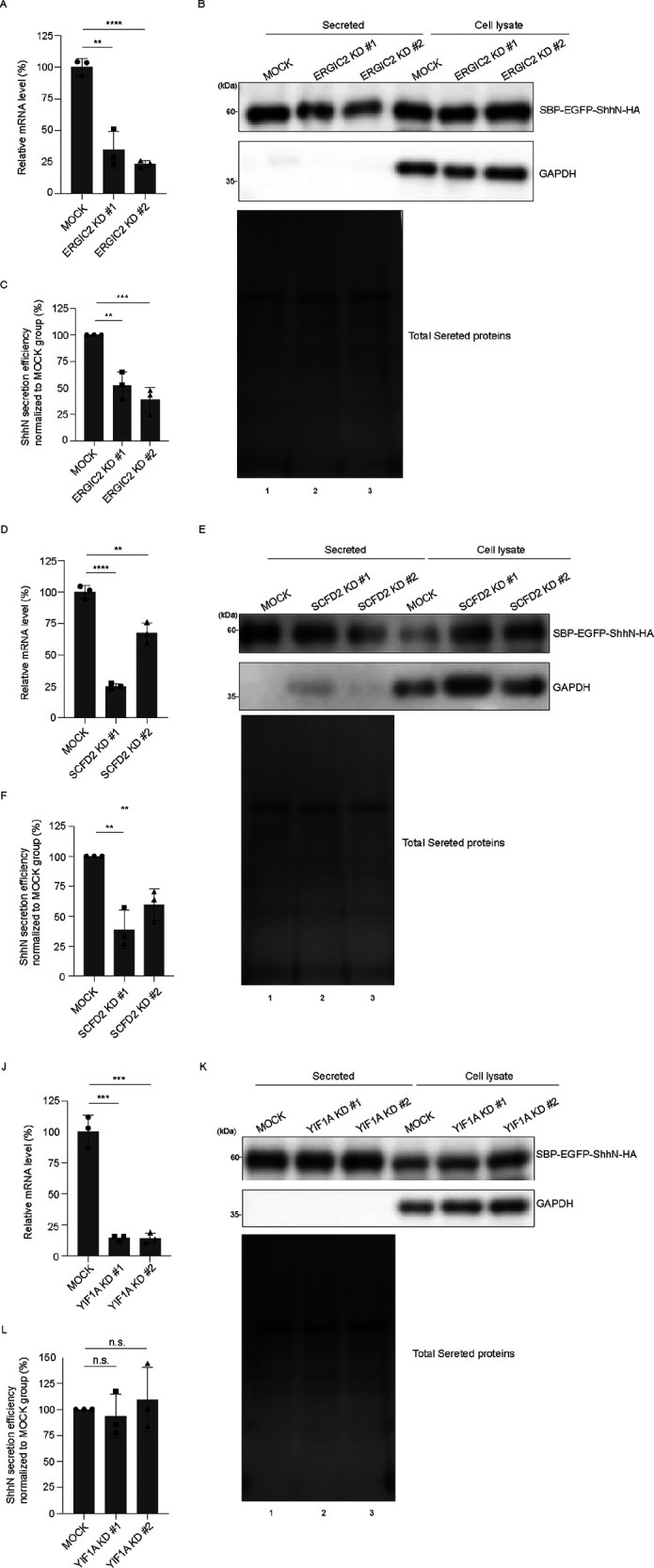
Knockdown of ERGIC2 and SCFD2 impairs the secretion of SBP-EGFP-ShhN, whereas YIF1A knockdown does not. (A, D, and J) HEK 293T cells were transfected with control siRNA or two different siRNAs against ERGIC2 (A), SCFD2 (D) or YIF1A (J). Seventy-two hours after transfection, the relative mRNA levels of indicated genes were analyzed by RT-qPCR (mean ± SD; *n* = 3). **P < 0.01, ***P < 0.001, ****P < 0.0001. (B, E, and K) HEK293T cells were transfected with control siRNA or two different siRNAs against ERGIC2 (B), SCFD2 (E) or YIF1A (K). Forty eight hours after transfection, cells were further transfected with plasmids encoding Str-KDEL_SBP-EGFP-ShhN-HA. Seventy two hours after knockdown, cells were incubated with biotin for 90 min. After biotin incubation, the level of SBP-EGFP-ShhN-HA in the medium and in cell lysates was analyzed by immunoblotting with anti-HA, and the level of total proteins in the medium was analyzed by silver staining. (C, F, and L) Quantification of the abundance of secreted SBP-EGFP-ShhN-HA 90 min after biotin treatment, normalized to the abundance detected in the cell lysate fraction (mean ± SD; *n* = 3). n.s., not significant, **P < 0.01, ***P < 0.001.

To assess the role of these three candidates in ShhN secretion, we performed knockdown experiments coupled with the Retention Using Selective Hook (RUSH) assay (Boncompain *et al.*, 2012). The RUSH plasmid of ShhN consists of: (1) ShhN (amino acid: 25 to 198) fused with streptavidin-binding peptide (SBP), enhanced green fluorescent protein (EGFP), and 3 × HA tag (SBP-EGFP-ShhN-HA); (2) an ER retention signal (Lys-Asp-Glu-Leu; KDEL) fused with streptavidin (str; str-KDEL). This system enables biotin-controlled synchronous secretion of ShhN. We utilized two different siRNAs against ERGIC2, SCFD2, or YIF1A to reduce their expression, respectively (Figure 3A, D, and J, and Supplemental Figure S2, A and B) and examined their effects on ShhN secretion. Secretion of SBP-EGFP-ShhN-HA was significantly reduced upon knockdown of ERGIC2 or SCFD2 compared with MOCK control (Figure 3, B and E, and quantifications in 3C and F), confirming that these two proteins contribute to Shh secretion. YIF1A knockdown did not impair ShhN secretion (Figure 3K and quantification in 3L). To confirm that the secretion defects were specifically caused by depletion of ERGIC2 and SCFD2 rather than off-target effects, we performed rescue experiments using siRNA-resistant constructs. We generated plasmids encoding Myc-tagged ERGIC2 and SCFD2, and then introduced siRNA-resistant mutations (ERGIC2^RS^-Myc and SCFD2^RS^-Myc) via site-directed mutagenesis. Co-transfection of ERGIC2^RS^-Myc or SCFD2^RS^-Myc significantly restored the secretion of SBP-EGFP-ShhN-HA in knockdown cells (Supplemental Figure S3, A and C, and quantifications in S3, B and D). These results confirmed that the impaired ShhN secretion observed upon ERGIC2 or SCFD2 depletion is indeed caused by the loss of these specific proteins.

### Defining the specific secretory step regulated by ERGIC2 and SCFD2

**FIGURE 4:**
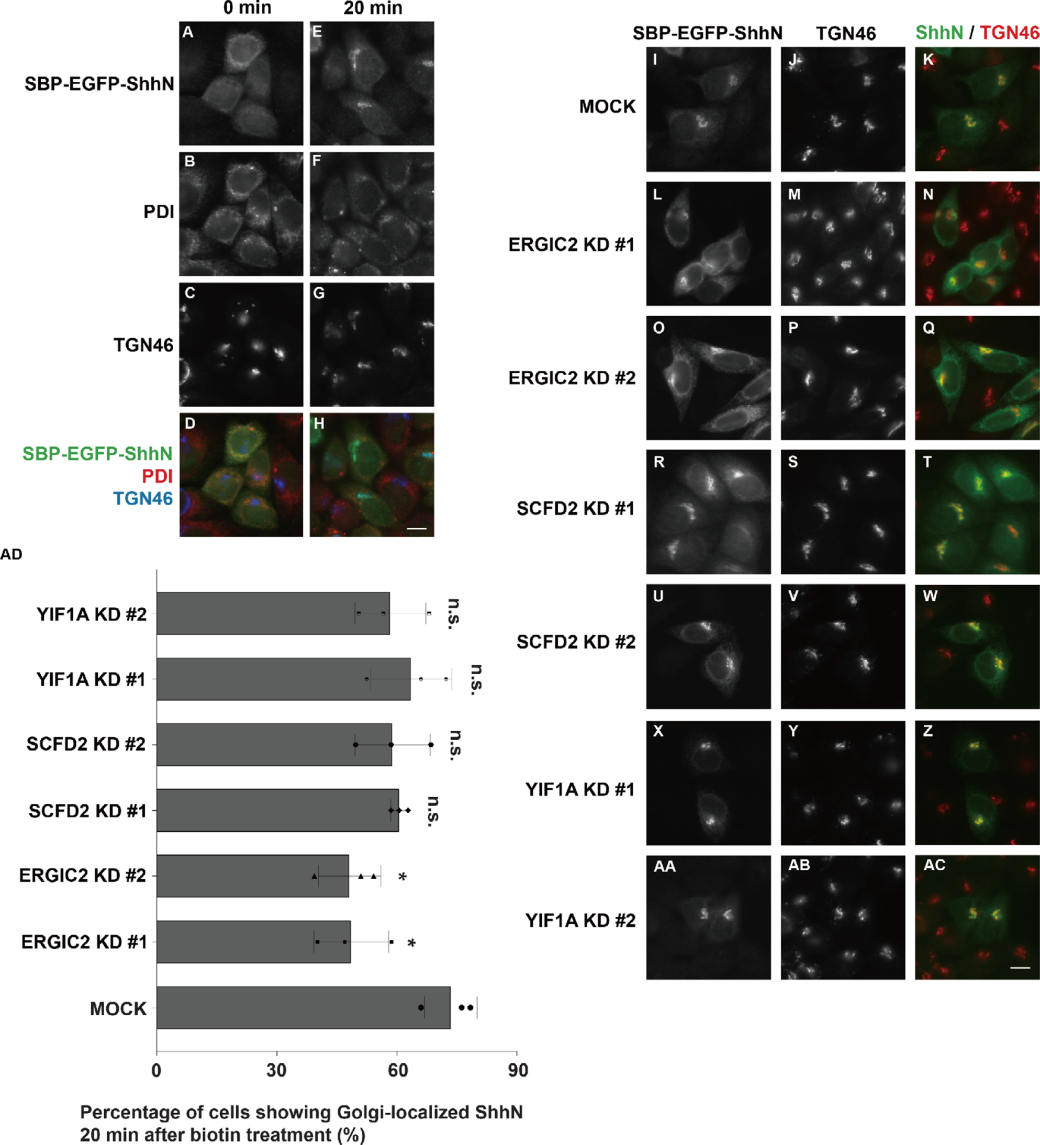
Knockdown of ERGIC2 impairs the ER-to-Golgi transport of SBP-EGFP-ShhN. (A–H) HeLa cells were transfected with plasmids encoding Str-KDEL_SBP-EGFP-ShhN. Twenty-four hours after transfection, cells were treated with biotin and incubated at 37°C for 20 min, and the localization of SBP-EGFP-ShhN was analyzed (Scale bar, 10 μm). (I–AC) HeLa cells were transfected with control siRNA (I–K) or two different siRNAs against ERGIC2 (L–Q), SCFD2 (R–W) or YIF1A (X-AC). Forty-eight hours after transfection, cells were transfected with plasmids encoding Str-KDEL_SBP-EGFP-ShhN. Seventy-two hours after knockdown, cells were treated with biotin and incubated at 37°C for 20 min, and the localization of SBP-EGFP-ShhN was analyzed (Scale bar, 10 μm). (AD) Quantifications of the percentage of cells showing Golgi-localized SBP-EGFP-ShhN signal after incubation with biotin for 20 min (mean ± SD; *n* = 3; >100 cells counted for each time point). n.s., not significant, *P < 0.05, **P < 0.01.

We next sought to define the specific secretory step regulated by these protein hits. We first examined their potential roles in ER-to-Golgi transport of ShhN using the RUSH system. In the absence of biotin, SBP-EGFP-ShhN was retained in the ER, where it colocalized with the ER marker protein disulfide isomerase (PDI; Figure 4, A–D). Twenty minutes after biotin treatment, most of the cells displayed a juxta-nuclear, Golgi-like distribution of SBP-EGFP-ShhN that colocalized with Golgi marker, TGN46 (Figure 4, E–H). Knockdown of ERGIC2 significantly reduced the proportion of cells showing Golgi-localized ShhN signal (Figure 4, I–Q and quantification in AD), indicating that ERGIC2 contributes to efficient ER export of Shh. Notably, ∼ 40-50% of cells still exhibited Golgi-localized ShhN after biotin treatment in the ERGIC2 knockdown group (Supplemental Figure 4, A–D), possibly reflecting incomplete protein depletion or compensatory mechanisms involving alternative trafficking factors, requiring further investigation.

To exclude the possibility that ERGIC2 knockdown indirectly affects Golgi integrity or globally slows ER-to-Golgi transport, we performed two control experiments. First, immunostaining of cis-Golgi marker GM130 and trans-Golgi marker TGN46 revealed comparable Golgi morphology between control and ERGIC2 knockdown cells (Supplemental Figure S4, A–L), indicating that ERGIC2 depletion does not grossly disrupt Golgi structure. Second, using another soluble cargo, insulin-like growth factor 2 (IGF2) -RUSH (Li *et al.*, 2023), as a control cargo, we found that ERGIC2 knockdown did not impair its ER-to-Golgi transport (Supplemental Figure S5, A–I and quantification in S5J), in contrast to the clear defect observed for ShhN (Figure 4, I–Q and quantification in AD). These results demonstrate that ERGIC2 functions in a cargo-selective manner rather than through global disruption of the secretory pathway. In contrast, SCFD2 knockdown did not significantly alter ER-to-Golgi transport of ShhN (Figure 4, R–W and quantification in AD). Similarly, YIF1A depletion had no detectable effect on this step (Figure 4X-AC and quantification in AD), consistent with its lack of impact on ShhN secretion (Figure 3, K–L).

**FIGURE 5:**
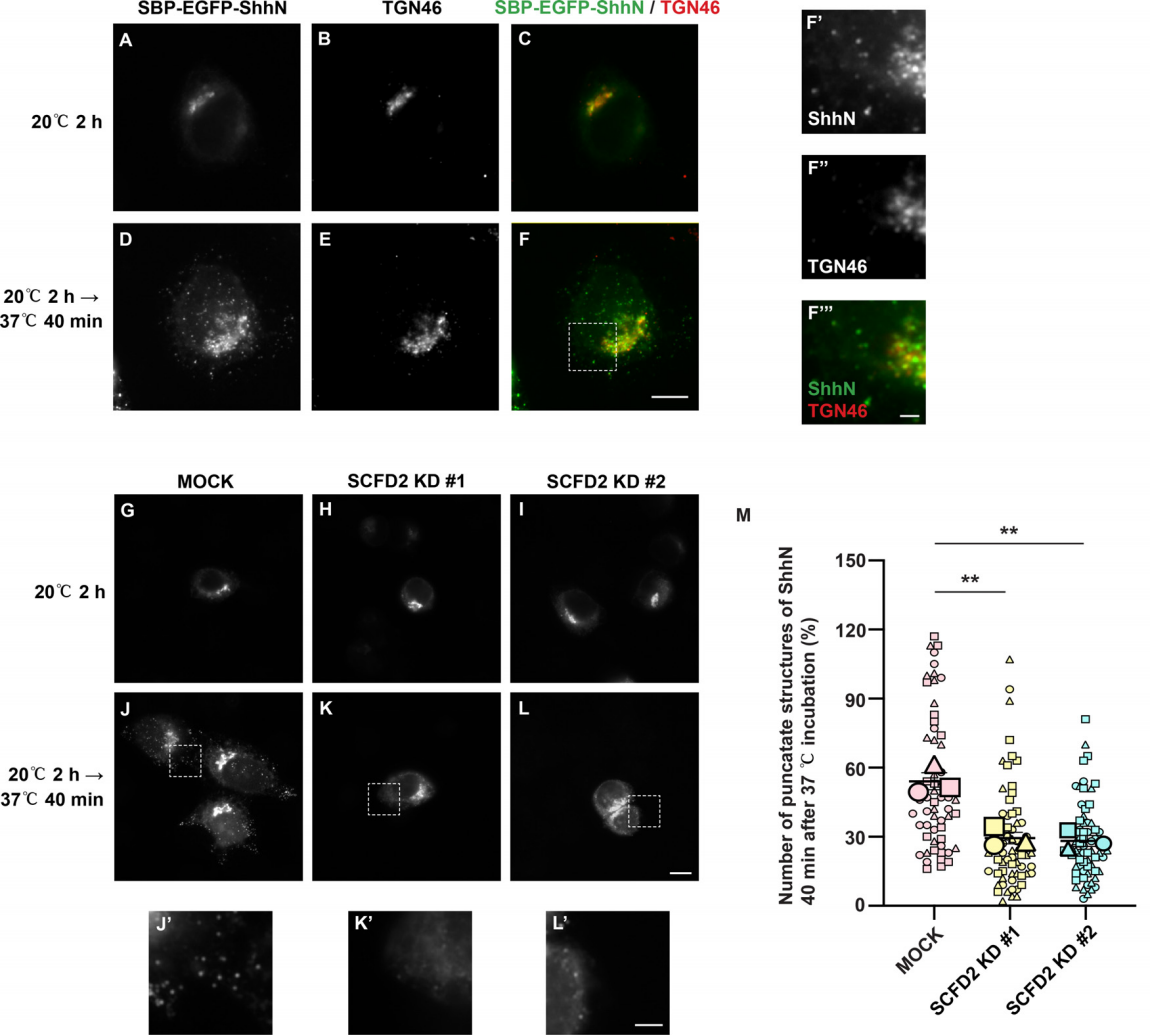
Knockdown of SCFD2 impairs the post-Golgi trafficking of SBP-EGFP-ShhN. (A–F) HeLa cells were transfected with plasmids encoding Str-KDEL_SBP-EGFP-ShhN. Twenty-four hours after transfection, cells were treated with biotin and incubated at 20°C for 2 h. Then the cells were incubated at 37°C for 0 min (A–C) or 40 min (D–F), and the localization of SBP-EGFP-ShhN and TGN46 were analyzed (Scale bar, 10 μm). The magnified views of the indicated area in panels F are shown in panels F’–F’’’ (Scale bar, 2 μm). (G–L) HeLa cells were transfected with control siRNA (G and J) or two different siRNAs against SCFD2 (H and I, and K and L). 48 h after transfection, cells were transfected with plasmids encoding Str-KDEL_SBP-EGFP-ShhN. Seventy-two hours after knockdown, cells were treated with biotin and incubated at 20°C for 2 h. Then the cells were incubated at 37°C for 0 min (G–I) or 40 min (J–L), and the localization of SBP-EGFP-ShhN was analyzed (Scale bar, 10 μm). The magnified views of the indicated area in panels J–L are shown in panels J’–L’ (Scale bar, 5 μm). (M) Quantification of the number of punctate structures containing SBP-EGFP-ShhN per cell 40 min after 37°C incubation (*n* = 3, mean ± SD, over 20 cells were quantified in each experimental group). ****P < 0.0001.

Since SCFD2 depletion did not affect the ER-to-Golgi transport of ShhN, we then investigated its potential involvement in post-Golgi trafficking. A temperature shift assay was employed to synchronize cargo export from the Golgi. HeLa cells transfected with RUSH construct of ShhN were incubated with biotin at 20°C to accumulate SBP-EGFP-ShhN in the Golgi, where it co-localized with the Golgi marker, TGN46 (Figure 5, A–C). The temperature was then shifted to 37°C to release cargo from the Golgi. Forty minutes after the shift, SBP-EGFP-ShhN displayed a punctate distribution, reflecting its incorporation into Golgi-derived transport vesicles (Figure 5, D–F, and magnified views in F’–F’’’). SCFD2 knockdown significantly reduced the number of SBP-EGFP-ShhN-positive puncta per cell compared with control cells (Figure 5, J–L, magnified views in J’–L’ and quantification in M). Collectively, these findings demonstrate distinct trafficking roles: ERGIC2 regulates ER-to-Golgi transport of ShhN, while SCFD2 mediates TGN export, both contributing ShhN secretion.

## DISCUSSION

The precise dissection of cargo-specific secretory pathways has long been constrained by a fundamental methodological challenge: distinguishing proteins that are merely near a cargo from those functionally co-incorporated into the transport vesicles. Although proximity-dependent biotinylation techniques, such as APEX2 and Proximity-Dependent Biotin Identification (BioID), have greatly advanced spatial proteomics, candidate lists derived from these methods are often confounded by abundant resident proteins of donor compartments, hindering the identification of genuine vesicle-associated transport factors (Roux *et al.*, 2012; Hung *et al.*, 2016). To overcome this challenge, we developed an integrated proximity biotinylation and in vitro vesicle formation assay. This strategy combines the spatial specificity of proximity labeling with the functional resolution of a cell-free vesicle budding reaction, enabling the selective enrichment of proteins that are co-packaged with a cargo into transport vesicles.

Applying this method to ShhN, we identified a high-confidence set of vesicle-associated interactors and validated ERGIC2 and SCFD2 as novel regulators that function at distinct steps of Shh secretion. Our functional analyses defined their roles: ERGIC2 is required for efficient ER-to-Golgi transport of ShhN, whereas SCFD2 is essential for its export from the TGN. The role of ERGIC2 is particularly significant given its known function as a COPII-associated protein in the transport of gap junction proteins (Guan *et al.*, 2022) and the dual roles of its yeast ortholog, Erv41, in both anterograde and retrograde trafficking at the ER-Golgi interface (Fuesler *et al.*, 2025). This indicates that the ERGIC2/Erv41 family plays an evolutionarily conserved yet functionally adaptable role in regulating early secretory cargo transport. As a member of the Sec1/Munc18 (SM) protein family, SCFD2 likely acts as a regulator of SNARE complex assembly (Eisemann *et al.*, 2020). This molecular activity is consistent with our observation that SCFD2 depletion reduces the number of ShhN-containing post-Golgi carriers, a phenotype that could reflect either a direct role in TGN vesicle formation or, in line with its canonical SM function, an indirect consequence of impaired vesicle fusion and subsequent turnover. Distinguishing between these possibilities will require further mechanistic investigation. The precise biochemical mechanisms of these newly identified factors remain to be fully elucidated.

The integration of these new regulators with the previously established SURF4-PG relay mechanism (Tang *et al.*, 2022) and the GRIA1-dependent TGN export pathway (Tang *et al.*, 2025) allows us to propose a more complete model for ShhN trafficking. SURF4 mediates the initial capture and COPII packaging of ShhN at the ER. ERGIC2 then promotes efficient ER export, possibly by stabilizing the cargo-receptor complex or promoting vesicle budding. Upon reaching the Golgi, PGs displace SURF4, which recycles back to the ER. At the TGN, GRIA1 recognizes and recruits ShhN through a PG-mediated interaction, while SCFD2, as an SM-family protein, likely regulates SNARE-complex assembly. Collectively, these components assemble into a coordinated machinery that packages ShhN into post-Golgi carriers destined for the plasma membrane. This multistep, factor-handoff mechanism ensures both the fidelity and efficiency of Shh secretion.

The integrated assay developed here holds significant potential for broader applications. Beyond Shh, it can be applied to map the vesicular interactomes of other clinically relevant cargo proteins, such as Wnt morphogens, growth factors, and disease-associated secreted proteins. It could also elucidate the machinery underlying polarized trafficking in neurons and epithelial cells. Extending this platform to specialized secretory granules, such as insulin granules and dense core vesicles, represents an important frontier. Since these granules differ from COPII and post-Golgi vesicles in their dependence on specific lipids, Ca²⁺, pH, and accessory proteins, adapting our platform will require supplementing the budding reaction with Ca²⁺ and adjusting the pH to better mimic the granule biogenesis environment, as well as replacing rat liver cytosol and donor membranes with those derived from relevant cell types to provide tissue-specific factors. This platform can be further optimized and extended in several ways. For instance, by including a direct comparison with APEX2 biotinylation alone to further validate vesicle-specific interactors, or by coupling organelle-specific fractionation to achieve higher spatial resolution, or by adapting it for high-throughput screening in combination with CRISPR-based gene editing to systematically identify genetic regulators of cargo-specific pathways (Kampmann *et al.*, 2015; Christopher *et al.*, 2021). Nevertheless, the use of 1 mM H₂O₂ for 1 minute, while a standard protocol for APEX2-based proximity labeling (Paek *et al.*, 2017; Kalocsay, 2019), could potentially induce cellular stress or artifacts. Future applications of this integrated platform to more peroxide-sensitive systems, such as peroxisomes, should consider optimizing H₂O₂ concentration (e.g., 0.5 mM or lower) and duration (e.g., 30 s), or exploring alternative proximity labeling enzymes with less reliance on peroxide, such as TurboID.

In conclusion, through the development of an integrated proximity labeling and in vitro vesicle reconstitution assay, we have identified and validated ERGIC2 and SCFD2 as functional components of the Shh secretory machinery, thereby advancing our molecular understanding of morphogen trafficking. Our findings not only provide a deeper mechanistic insight into Shh secretion but also open new avenues for investigating related developmental diseases and cancers. The methodology established herein offers a powerful tool for both fundamental cell biology research and translational applications, enabling the systematic identification of novel therapeutic targets in diseases characterized by secretory dysregulation.

## MATERIALS AND METHODS

Request a protocol through *Bio-protocol*

### Constructs, reagents, cell culture, transfection, and immunofluorescence

HeLa and HEK293T cell lines were kindly provided by the University of California-Berkeley Cell Culture Facility and were confirmed by short tandem repeat profiling. All cell lines tested negative for Mycoplasma contamination. All cell lines were cultured in Dulbecco's Modified Eagle Medium (DMEM) containing 10% fetal bovine serum and 1% penicillin streptomycin mix (Invitrogen).

The cDNA encoding mouse ShhN, human SURF4, human IGF2, human ERGIC2, and human SCFD2 were ordered from BGI (Beijing, China). The plasmids encoding C-terminal 3xHA-tagged ShhN, C-terminal 3xMyc-tagged SURF4, ShhN-HA-APEX2, Str-KDEL_SBP-EGFP-ShhN (aa: 25–198), Str-KDEL_SBP-EGFP-IGF2, C-terminal 3xMyc-tagged ERGIC2 and C-terminal 3xMyc-tagged SCFD2 were generated by standard molecular cloning procedures. The siRNA-resistant mutations, ERGIC2^RS^-Myc and SCFD2^RS^-Myc, were generated using site-directed mutagenesis. The N-terminus of the SBP-EGFP tag is followed by a signal sequence derived from IL-2 (Boncompain *et al.*, 2012).

siRNAs against ERGIC2, SCFD2, and YIF1A were purchased from Gene Create (Wuhan, China). The target sequence of the two siRNAs against human ERGIC2 is GTTTCAGACTTGGATCCTATA and GCCATGAAGTGTCAATATG, respectively. The target sequence of the two siRNAs against human SCFD2 is GGAGATCCTACGGGACATCAT and ATGATGTGATGGTTAACATGATA, respectively. The target sequence of the two siRNAs against human YIF1A is GATGGCACTGGGCATTCAGAA and GGCGCTCATGTACTTCATTGT, respectively.

The commercial antibodies were rabbit anti-HA (Cell Signaling, Catalogue number 3724), mouse anti-Myc (Cell Signaling, Catalogue number 2276), mouse anti-GAPDH (Proteintech, Catalogue number 60004-1), sheep anti-TGN46 (BIO-RAD, Catalogue number AHP500G), mouse anti-β-actin (Proteintech, Catalogue number 60008-1-Ig), rabbit anti-Calnexin (Abcam, Catalogue number ab22595), mouse anti-GM130 (BD biosciences, Catalogue number 610823), rabbit anti-ERGIC2 (Proteintech, Catalogue number 11927-1-AP) and rabbit anti-SCFD2 (Proteintech, Catalogue number 13375-1-AP). Mouse anti-SURF4 antibodies were kindly provided by Prof. Xiaowei Chen (Peking University, China). Rabbit anti-SEC22B antibodies for the immunoblot analyses were kindly provided by Prof. Randy Schekman (University of California, Berkeley).

Transfection of siRNA or DNA constructs into HeLa cells, or HEK293T cells, and immunofluorescence were performed as described previously (Tang *et al.*, 2022). Images were acquired with an Eclipse Ti Motorized Inverted Fluorescence Microscope (Nikon) equipped with an Andor Zyla 4.2 sCMOS camera (Andor Technology) or Leica SP8 Confocal Laser Scanning Microscope (Leica).

### RUSH assay

RUSH assays were performed by treating HeLa cells transfected with plasmids encoding Str-KDEL_SBP-EGFP-ShhN in a complete medium containing 40 μM biotin (Sigma-Aldrich) and 100 ng/μl cycloheximide (Sigma-Aldrich) at the indicated temperature for the indicated time. Cells were then fixed by 4% paraformaldehyde and mounted on glass slides with ProLong Gold Antifade Mountant with DAPI (Invitrogen) for microscope analysis. All image analyses were performed using Fiji (ImageJ) software. For quantification of the percentage of the cells showing Golgi-localized ShhN or IGF2 signal, SBP-EGFP-ShhN or SBP-EGFP-IGF2-positive cells were randomly selected without preselection. To ensure unbiased selection, cells were first visualized under bright-field illumination to identify the field, and then EGFP-positive cells within that field were counted consecutively. Transfection efficiency was comparable across conditions and did not require normalization. The number of punctate structures was quantified using the “Analyze Particles” function in Fiji (ImageJ). Before analysis, a consistent background subtraction was applied to all images. A fixed noise tolerance (prominence) value was used to robustly distinguish specific signals from background, and maxima at the image edges were excluded.

To analyze the secretion of ShhN, HEK293T cells transfected with plasmids encoding Str-KDEL-SBP-EGFP-ShhN-HA were treated with 100 ng/μl cycloheximide and 40 μM biotin in medium without FBS addition for the indicated time. Then the secreted proteins were collected by Trichloroacetic acid (TCA) precipitation. The cells were collected and lysed by HKT buffer (100 mM KCl, 20 mM HEPES, pH 7.2, 0.5% Triton X-100). The precipitated proteins and cell lysates were analyzed by immunoblotting.

### Proximity, biotinylation assay, and in vitro vesicle formation assay

For proximity-dependent biotinylation, HEK293T cells were transfected with plasmids encoding ShhN-HA-APEX2. Twenty-four hours after transfection, the cells were incubated with 500 μM biotin tyramide at 37°C for 1 h to allow substrate loading. Proximity labeling was then initiated by adding 1 mM hydrogen peroxide (H₂O₂) to the medium and incubating for exactly 1 min at room temperature. The reaction was immediately quenched by replacing the medium with a freshly prepared, ice-cold quenching solution containing 10 mM sodium ascorbate, 5 mM Trolox, and 10 mM sodium azide in phosphate-buffered saline (PBS). The cells were then incubated in the quenching solution at 4°C for 10 min in the dark to ensure complete termination of APEX2 activity. Following quenching, the cells were harvested to perform an in vitro vesicular release assay.

In vitro vesicular release assays were performed as described previously (Tang *et al.*, 2020). Briefly, 24 h after transfection with ShhN-HA-APEX2, HEK293T cells were permeabilized with 40 μg/ml digitonin on ice. The semi-intact cells were washed and incubated with rat liver cytosol in the presence of GTP and an ATP regeneration system, with or without the dominant-negative Sar1A (H79G), at 32°C for 1 h. The released vesicles were isolated by centrifugation at 14,000 × g and subsequently lysed in Tris-based buffer containing 1% (vol/vol) Triton X-100. Then the lysates were incubated with NeutrAvidin Agarose Resin (Thermo Fisher Scientific, Catalogue number 29201) with mixing at 4°C overnight. The bound proteins were collected and analyzed by immunoblotting or Coomassie blue staining.

### Quantitative real-time PCR (qPCR)

Total RNA was extracted from HEK 293T cells by PureLink RNA Mini Kit (Thermo Fisher Scientific, Catalogue number 12183018A), and 1 μg RNA was reverse-transcripted into cDNA using Applied BiosystemsTM High-Capacity cDNA Reverse Transcription Kit (Thermo Fisher Scientific, Catalogue number 4374966). Quantitative PCR reaction was performed using LightCycler 480 SYBR Green I Master kit (Roche, Catalogue number 04707516001). All the procedures were conducted according to the manufacturer's guide. Genes are calculated using the 2–∆∆Ct method and normalized to Gadph. Primers targeting human Ergic2 (fwd: GGACCATAACACAAGCATGAC, rvs: TCAGGAACCTTCGGAAAGGC), human Scfd2 (fwd: AAACACCCACAGACTGCCAA, rvs: AACGGACATTGCTGACTCCC), and human Yif1a (fwd: ATGGCTTATCACTCGGGCTAC, rvs: CCGCTTGTGTCATCGAAGAGG) were used.

### Label-free quantitative mass spectrometry and bioinformation analysis

Label-free quantitative mass spectrometry was performed as described previously (Tang *et al.*, 2022). Briefly, following proximity biotinylation assay and in vitro vesicular release assay, the bound proteins were resolved by SDS–PAGE and visualized by Coomassie blue staining. The gel regions containing proteins of interest were excised, destained, reduced, alkylated, and digested in-gel with sequencing-grade trypsin (Promega, number V511A). Peptides were extracted, desalted, and analyzed by liquid chromatography–tandem mass spectrometry (LC-MS/MS). Protein identification and label-free quantification were performed using Proteome Discoverer software, with interacting proteins distinguished by comparing their spectral intensities between +RLC and −RLC samples.

Protein intensities were first normalized against the total ion current (TIC) of each sample to obtain relative quantitative values. The Venn diagram was generated using the Vennerable R package (version 3.1.0.9). Gene Ontology (GO) enrichment analysis for cellular components was performed with the clusterProfiler R package (version 4.12.6). Differentially expressed biotinylated proteins were identified using Perseus software (version 1.6.6.0). Significance was assessed using Student's *t* test, and *p* values were corrected via permutation-based false discovery rate (FDR) adjustment (q-value). Visualization of results, including volcano plots, GO enrichment dot plots, and cumulative abundance curves, was carried out using the ggplot2 R package (version 3.5.12).

## Supporting information





## Abbreviations used:

APEX2: Ascorbate Peroxidase 2
ATPrS: ATP regeneration system
BioID: Proximity-Dependent Biotin Identification
COP: Coat Protein Complex
DMEM: Dulbecco's Modified Eagle Medium
EGFP: Enhanced Green Fluorescent Protein
ER: Endoplasmic Reticulum
ERGIC2: ER-Golgi Intermediate Compartment Protein 2
Erv41: ER-derived vesicle protein 41
FDR: False Discovery Rate
GRIA1: Glutamate receptor 1
GO: Gene Ontology
IGF2: Insulin-Like Growth Factor 2
KDEL: Lys-Asp-Glu-Leu (ER retention signal)
LC-MS/MS: Liquid Chromatography-Tandem Mass Spectrometry
PBS: Phosphate-Buffered Saline
PDI: Protein Disulfide Isomerase
PG: Proteoglycan
qPCR: Quantitative Reverse Transcription Polymerase Chain Reaction
RLC: Rat Liver Cytosol
RUSH: Retention Using Selective Hook
SBP: Streptavidin-Binding Peptide
SCFD2: Sec1 Family Domain Containing 2
Shh: Sonic hedgehog
SM: Sec1/Munc18
SNARE: Soluble N-ethylmaleimide-sensitive factor attachment protein receptor
SURF4: Surfeit locus protein 4
TCA: Trichloroacetic Acid
TIC: Total Ion Current
YIF1A: Yip1 Interacting Factor Homolog A.
