# Supplementary material for: An integrated proximity labeling and vesicle reconstitution assay identifies novel regulators of Sonic hedgehog secretion
Source: Mol Biol Cell. 2026 May 28;37(6):ar59. doi: 10.1091/mbc.E25-12-0627 (PMC13229121; doi:10.1091/mbc.E25-12-0627)
Supplement: Supplementary file 1 [file mbc-37-ar59-s001.pdf]

Supplemental Materials

Molecular Biology of the Cell

Song *et al*.

SI Appendix Figure S1-5

Figure S1

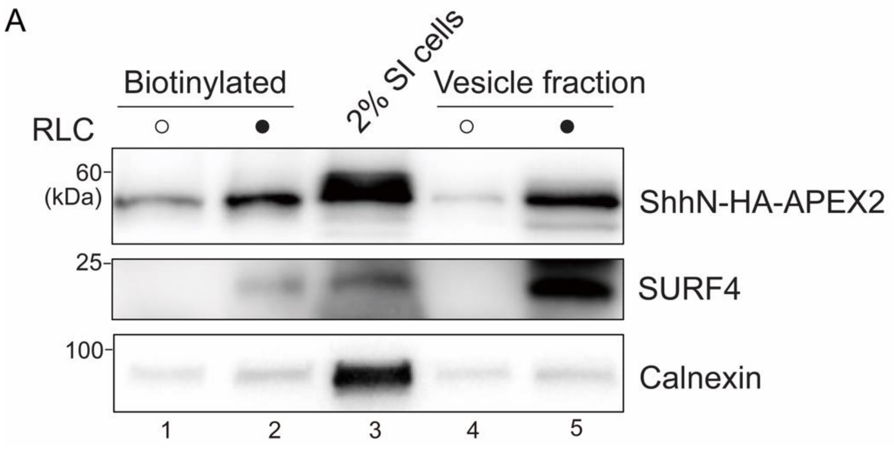

Figure S1. Proximity biotinylation and vesicle formation assay. (A) HEK293T cells were transfected with plasmid encoding ShhN-HA-APEX2. 24 h after transfection, proximity biotinylation and vesicle formation assay were performed in absence or presence of RLC. The biotinylated proteins in the vesicles were analyzed by SDS-PAGE and immunoblotting using anti-HA, anti-SURF4 or anti-Calnexin antibodies.

Figure S2

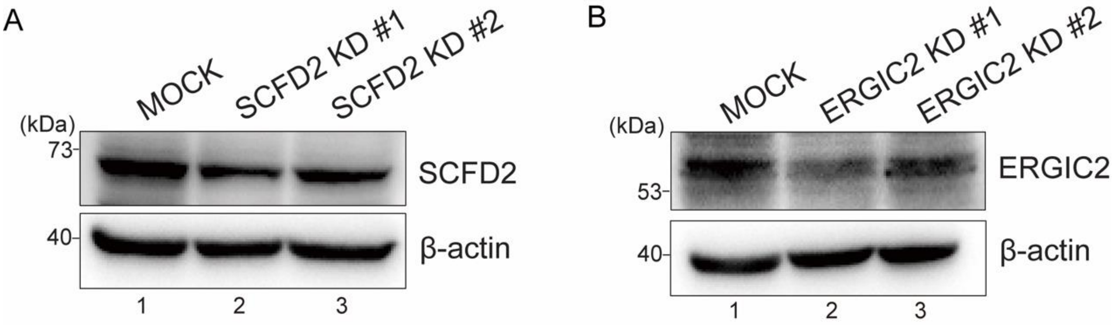

Figure S2. Knockdown efficiency of ERGIC2 and SCFD2 siRNAs assessed by immunoblotting. (A) HEK293T cells were transfected with control siRNA or two different siRNAs against SCFD2 (A) or ERGIC2 (B). 72 h after transfection, the relative levels of indicated proteins were analyzed by immunoblotting.

Figure S3

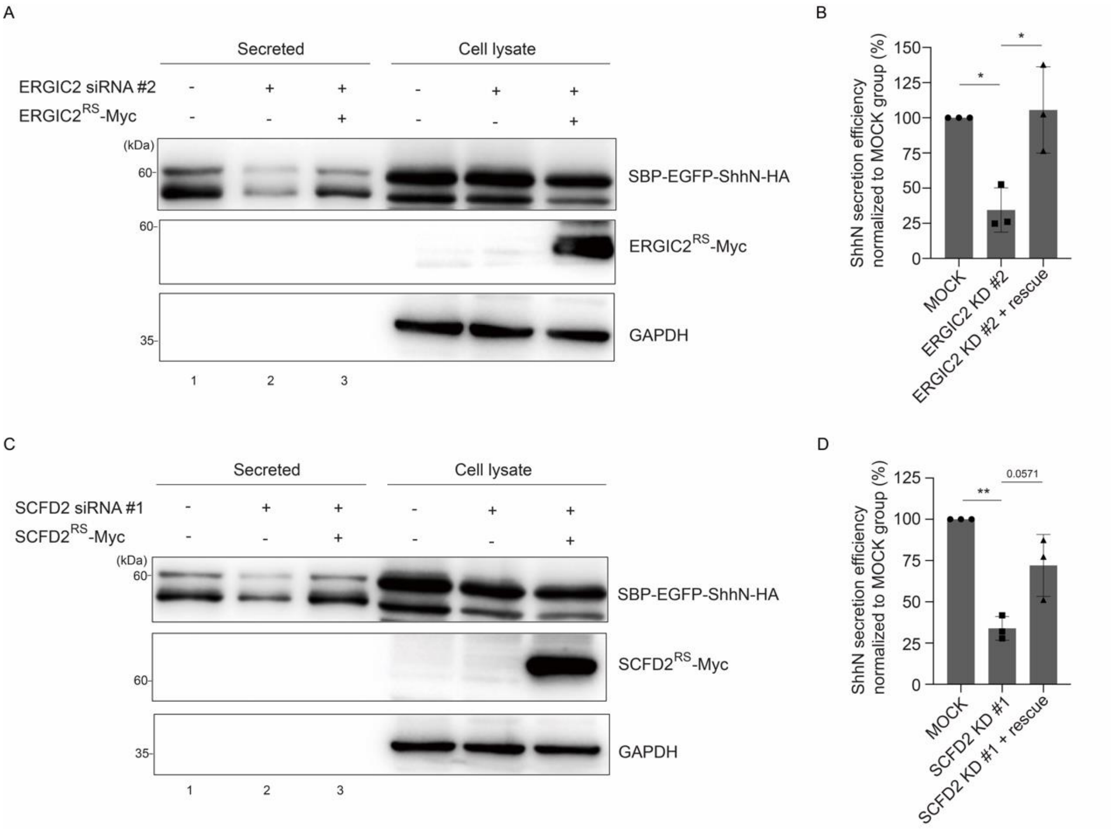

Figure S3. Expressing the siRNA resistant ERGIC2 and SCFD2 recues the secretion defects of ShhN in ERGIC2 and SCFD2 knockdown cells. (A and C) HEK293T cells were transfected with control siRNA or two different siRNAs against ERGIC2 (A) or SCFD2 (C). 48 h after transfection, cells were further transfected with indicated plasmids. 72 h after knockdown, cells were incubated with biotin for 90 min. After biotin incubation, the level of SBP-EGFP-ShhN-HA in the medium and in cell lysates was analyzed by immunoblotting with anti-HA. (B and D) Quantification of the abundance of secreted SBP-EGFP-ShhN-HA 90 min after biotin treatment, normalized to the abundance detected in the cell lysate fraction (mean ± SD; n = 3). *P < 0.05, **P < 0.01.

Figure S4

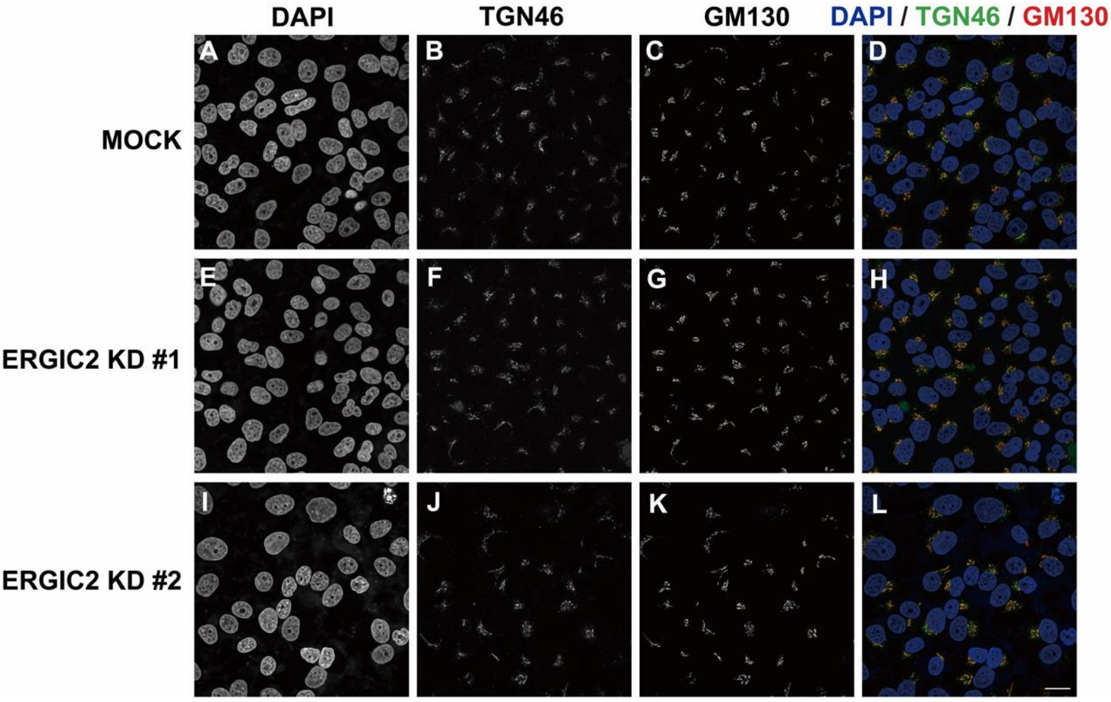

Figure S4. Knockdown of ERGIC2 didn't affect Golgi morphology. (A) HeLa cells were transfected with NC siRNA (A-D) or two different siRNAs against ERGIC2 (E-L). 72 h after knockdown, the localization of TGN46 and GM130 was analyzed (*Scale bar, 20 μm*).

Figure S5

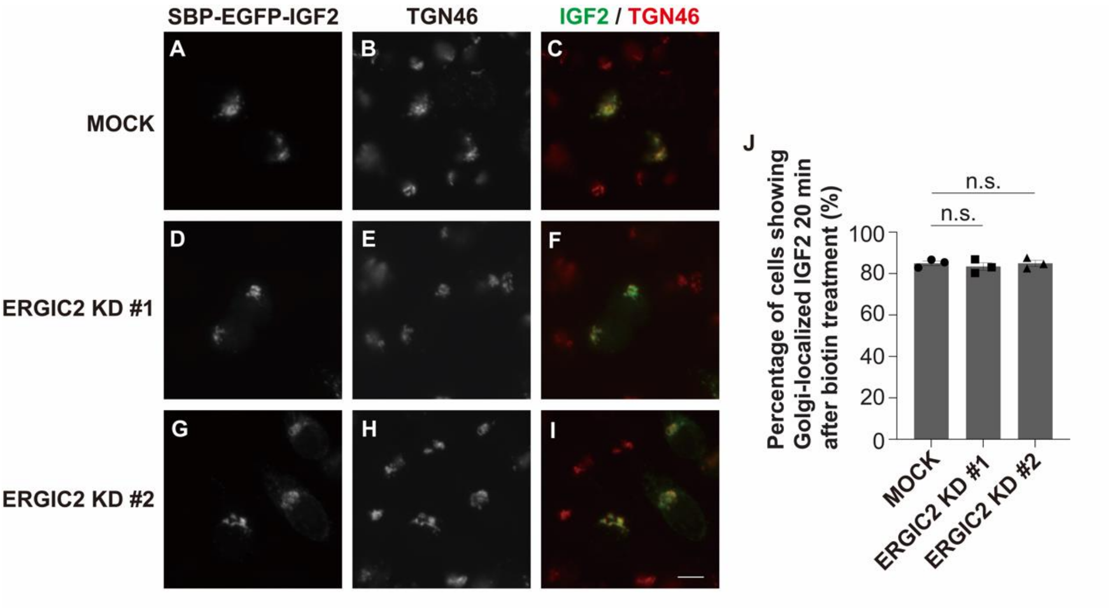

Figure S5. Knockdown of ERGIC2 didn't impair ER export of IGF2. (A-I) HeLa cells were transfected with control siRNA (A-C) or two different siRNAs against ERGIC2 (D-I). 48 h after transfection, cells were transfected with plasmids encoding Str-KDEL_SBP-EGFP-IGF2. 72 h after knockdown, cells were treated with biotin and incubated at 37 °C for 20 min, and the localization of SBP-EGFP-IGF2 was analyzed (*Scale bar,* 10 μm). (J) Quantification of the percentage of cells showing Golgi-localized SBP-EGFP-IGF2 signal after incubation with biotin for 20 min (mean ± SD; n = 3; >100 cells counted for each time point). n.s., not significant.
